# A randomized controlled trial protocol comparing the feeds of fresh versus frozen mother’s own milk for preterm infants in the NICU

**DOI:** 10.1186/s13063-019-3981-4

**Published:** 2020-02-11

**Authors:** Huiqing Sun, Yun Cao, Shuping Han, Rui Cheng, Ling Liu, Jiangqin Liu, Shiwen Xia, Jiajie Zhang, Zhankui Li, Xiuyong Cheng, Chuanzhong Yang, Xinnian Pan, Long Li, Xin Ding, Rensheng Wang, Mingyuan Wu, Xiaoying Li, Liping Shi, Falin Xu, Fengqin Yu, Jiahua Pan, Xiaolan Zhang, Li Li, Jie Yang, Mingxia Li, Changhong Yan, Qi Zhou, Jiao Lu, Mou Wei, Laishuan Wang, Ling Yang, Xiang Y. Ye, Sharon Unger, Foteini Kakulas, Shoo K. Lee

**Affiliations:** 1 Department of Neonatology, Children’s Hospital affiliated to Zhengzhou University, Henan Children’s Hospital, Zhengzhou Children’s Hospital, 33 Longhuwaihuan Road, Zhengzhou, 450018 Henan China; 2 Department of Neonatology, Children’s Hospital of Fudan University, 399 Wanyuan Road, Minhang District, Shanghai, 201102 Shanghai China; 3 Department of Pediatrics, The Affiliated Obstetrics and Gynecology Hospital of Nanjing Medical University, Nanjing Maternity and Child Health Care Hospital, 123 Tianfei Lane, Mochou Road, Qinhuai District, Nanjing, 210004 Jiangsu China; 4 Department of Neonatology, Children’s Hospital of Nanjing Medical University, 72 Guangzhou Road, Gulou District, Nanjing, 210008 Jiangsu China; 5 Department of Neonatology, Guizhou Maternity and Child Health Care Hospital, 63 Ruijin South Road, Guiyang, 530003 Guizhou China; 6 Department of Neonatology, Shanghai First Maternity and Infant Hospital, 2699 Gaoke West Road, Pudong New Area, Shanghai, 201204 China; 7 Department of Neonatology, The Women and Children’s Health-Care Hospital of Hubei Province, 745 Wuluo Road, Jiedaokou, Hongshan District, Wuhan, 430070 Hubei China; 8 Department of Neonatology, Henan Provincial People’s Hospital, 7 Weiwu Road, Zhengzhou, 450003 Henan China; 9 Department of Neonatology, Northwest Women and Children Hospital, 1616 Yanxiang Road, Qujiang New District, Xian, 710061 Shanxi China; 10 Department of Neonatology, The first affiliated hospital of Zhengzhou University Zhengzhou, 1 East Jianshe Road, Zhengzhou, 450052 Henan China; 11 Department of Neonatology, Shenzhen Maternity and Child Healthcare Hospital, 2004 Hongjing Road, Futian District, Shenzhen, 518017 Guangdong China; 12 Department of Neonatology, Maternal and Child Health Hospital of Guangxi Zhuang Autonomous Region, Nanning, Guangxi China; 13 Department of Neonatology, Xinjiang Uiger Municipal People’s Hospital, Tianchi Road, Urumqi, 830000 Xinjiang China; 14 Department of Neonatology, Children’s Hospital of Soochow University, 92 Zhongnan Street, SIP, Suzhou, 215025 Jiangsu China; 15 Department of Neonatology, Xiamen Children’s Hospital, 92-98 Yibin Road, Huli District, Xiamen, 361006 Fujian China; 16 Department of Neonatology, Women’s Hospital School of Medicine Zhejiang University, 1 Xueshi Road, Hangzhou, 31006 Zhejiang China; 17 Department of Neonatology, Qilu Children’s Hospital of Shandong University, 430 Jingshi Road, Lixia District, Jinan, 250022 Shandong China; 18 Department of Neonatology, Children’s Hospital School of Medicine Zhejiang University, 3333 Binsheng Road Binjiang District, Zhejiang, 310003 Hangzhou China; 19 Department of Neonatology, The Third Affiliated Hospital of Zhengzhou University, 7 Kangfuqian Street, Zhengzhou, 450052 Henan China; 20 Department of Neonatology, Women and Children Hospital of Zhengzhou, 41 Jinshui Road, Zhengzhou, 450012 Henan China; 21 Department of Neonatology, Anhui Provincial Hospital, 17 Qijiang Road, Hefei, 230001 Anhui China; 22 Department of Neonatology, Xianmen Humanity Hospital, 3777 Xianyue Road, Xiamen, 361000 China; 23 Department of Neonatology, Children’s Hospital of Capital Institute of Pediatrics, 2 Yabao Road, Chaoyang District, Beijing, 100020 China; 24 Department of Neonatology, Guangdong Women and Children Hospital, Guangzhou Medical University, 521-523, Xing Nan Road, Panyu, Guangzhou, 510000 China; 25 Department of Neonatology, First Affiliated Hospital of Xinjiang Medical University, 137 Road, Urumqi, 830054 Xinjiang China; 26 Department of Neonatology, Jiangxi Children’s Hospital, 122 Yangming Road, Nanchang, Jiangxi China; 27 Department of Neonatology, Shanghai General Hospital and Shanghai Jiaotong University, University 650, New Songjiang Road, Song Jiang, Shanghai, 201600 China; 28 Department of Neonatology, Guangzhou Women and Children’s Medical Center, 9 Jinsui Road, Tianhe District, Guangzhou, 510623 Guangdong China; 29 Department of Neonatology, Children’s Hospital of Hainan Province, 75 South Longkun Road, Haikou, 570206 Hainan Province China; 30 Maternal-Infant Care Research Centre, Mount Sinai Hospital, 700 University Avenue Rm 8-500, Toronto, ON M5G 1X6 Canada; 31 Department of Paediatrics, Sinai Health System, 600 University Avenue, Room 19-2310, Toronto, Ontario M5G 1X5 Canada; 32 Medical School, Faculty of Health and Medical Sciences, The University of Western Australia (M570), School of Medicine and Pharmacology, 35 Stirling Highway, 6009 Perth, Crawley, Western Australia Australia; 33 Departments of Pediatrics, Obstetrics & Gynecology, and Dalla Lana School of Public Health, University of Toronto, Toronto, Ontario Canada

**Keywords:** Breast milk, Preterm, Necrotizing enterocolitis, Neonatal intensive care unit (NICU)

## Abstract

**Background:**

Necrotizing enterocolitis (NEC) is the leading cause of death among preterm infants born at < 30 weeks’ gestation. The incidence of NEC is reduced when infants are fed human milk. However, in many neonatal intensive care units (NICUs), it is standard practice to freeze and/or pasteurize human milk, which deactivates bioactive components that may offer additional protective benefits. Indeed, our pilot study showed that one feed of fresh mother’s own milk per day was safe, feasible, and can reduce morbidity in preterm infants. To further evaluate the benefits of fresh human milk in the NICU, a randomized controlled trial is needed.

**Methods:**

Our prospective multicenter, double-blinded, randomized, controlled trial will include infants born at < 30 weeks’ gestation and admitted to one of 29 tertiary NICUs in China. Infants in the intervention (fresh human milk) group (*n* = 1549) will receive at least two feeds of fresh human milk (i.e., within 4 h of expression) per day from the time of enrollment until 32 weeks’ corrected age or discharge to home. Infants in the control group (n = 1549) will receive previously frozen human milk following the current standard protocols. Following informed consent, enrolled infants will be randomly allocated to the control or fresh human milk groups. The primary outcome is the composite outcome mortality or NEC ≥ stage 2 at 32 weeks’ corrected age, and the secondary outcomes are mortality, NEC ≥ stage 2, NEC needing surgery, late-onset sepsis, retinopathy of prematurity (ROP), bronchopulmonary dysplasia (BPD), weight gain, change in weight, increase in length, increase in head circumference, time to full enteral feeds, and finally, the number and type of critical incident reports, including feeding errors.

**Discussion:**

Our double-blinded, randomized, controlled trial aims to examine whether fresh human milk can improve infant outcomes. The results of this study will impact both Chinese and international medical practice and feeding policy for preterm infants. In addition, data from our study will inform changes in health policy in NICUs across China, such that mothers are encouraged to enter the NICU and express fresh milk for their infants.

**Trial registration:**

Chinese Clinical Trial Registry; #ChiCTR1900020577; registered January 1, 2019; http://www.chictr.org.cn/showprojen.aspx?proj=34276

## Background

Necrotizing enterocolitis (NEC) is a severe inflammatory disorder of the intestine that primarily affects very low birth weight (< 1500 g) or very preterm infants (≤ 32 weeks’ gestation); it is also the leading cause of death in the neonatal intensive care unit (NICU) [1, 2]. Between May 1, 2015, and April 28, 2018, we found that the incidence of mortality in infants born at < 30 weeks’ gestation at 25 hospitals in China was 25%, and the incidence of NEC was 6.2% [Siyuan Jiang, MD, written communication, October 2018]. Elsewhere, the overall mortality from NEC was reported to range from 15 to 30%, with more severe cases of NEC resulting in a higher mortality rate. For infants who survive, a significant risk exists for long-term complications and morbidity, including impaired neurodevelopment [1, 2].

Currently, no known effective treatment exists for NEC. Infants are initially managed medically through bowel rest (cessation of oral feeds), abdominal decompression, administration of broad-spectrum antibiotics, and provision of supportive care including parenteral nutrition, ventilator support, and blood transfusions as necessary. Should bowel perforation occur, then surgery is required to remove damaged tissue and gas build up through laparotomy, primary peritoneal drainage, or both [1, 2].

The World Health Organization and others have advocated for the use of mother’s own milk to prevent NEC [3–5]. Human milk contains both nutritional components (e.g., proteins, amino acids, fats, carbohydrates, vitamins, and minerals) and bioactive components (e.g., live immune cells, cytokines, hormones, and growth factors) that have antimicrobial and anti-inflammatory properties [6, 7]. More recently, Hassiotou (now Kakulas) et al. [8] showed that human milk is a rich source of pluripotent stem cells that, when ingested by the infant, enter the blood stream and are incorporated into the major organ systems of the infant. Although their exact function still remains unknown, breast milk stem cells possibly benefit the infant through actively participating in growth and/or regeneration. The current evidence strongly suggests that fresh human milk may have a significant protective effect against both infection and NEC [9].

When the mother’s own milk is not available, the next best option is donor human milk, and the last option, formula. The use of formula in low birth weight or very preterm infants was reported to increase the risk of developing NEC when compared with donor human milk [10]. Although human milk, either mother’s own or donor milk, appears to be the best feeding source for the nutrition, immunoprotection, and development of the preterm infant, current practices related to the handling and use of human milk in the NICU raise questions about whether we are deriving the maximum benefit from its use. Currently, in many NICUs worldwide, milk expressed by mothers of preterm infants is frozen and stored prior to use. When the infant requires oral feeds, frozen milk is defrosted and warmed, fortifier is added as needed, and the milk is then fed to the infant. Freezing human milk at either − 20 °C or − 80 °C and for longer times decreases the energy content and amount and bioactivity of several important components of human milk [11, 12], including the fat, carbohydrates, secretory immunoglobulin A, lactoperoxidase, lysozyme, antibacterial factors, and antioxidants [11–18]. In addition, defrosted human milk does not contain any live stem cells because they have a half-life of approximately 4 h and die when human milk is frozen and/or heated [8].

The current NICU human milk feeding procedure exists as a means of ensuring that infants have consistent access to their mother’s milk even if the mother is not able to spend time in the NICU. The process also allows for stricter quality and infection control, computerized inventory, and monitoring via electronic health records. However, this same process deprives infants of the benefits of the cellular content of human milk, including immune cells, stem cells, and the protective effects of the plethora of bioactive immunological components [8].

We recently conducted a pilot study entitled “Testing the feasibility and safety of feeding preterm infants fresh mother’s own milk in the NICU: a pilot study,” where we evaluated the feasibility and safety of providing preterm infants (born at < 30 weeks’ gestation) with at least one daily feed of fresh mother’s own milk, within 4 h of expression, until they reached 32 weeks’ corrected age [19]. The results showed that one feed of fresh human milk per day was both safe and feasible in the NICU, and it reduced morbidity among infants of < 30 weeks’ gestation.

For this randomized controlled trial, our primary objective is to evaluate the impact of feeding infants born at < 30 weeks’ gestation fresh unprocessed mother’s own milk within 4 h of expression on the primary outcome, the composite of mortality, or NEC ≥ stage 2 at 32 weeks’ corrected age. Our hypothesis is that the rate of the primary outcome will be lower for preterm infants who are fed at least two feeds of fresh mother’s own milk per day from the time of enrollment until 32 weeks’ corrected gestational age than for the control group infants who are fed human milk following the standard NICU human milk handling and feeding procedures. We will test our hypothesis using a randomized controlled trial with parallel groups, a one-to-one allocation ratio, and a singularity framework.

## Methods/Design

### Study design

Our study will be a prospective, multicenter, blinded, randomized, controlled trial (RCT) designed in accordance with the Standard Protocol Items: Recommendations for Interventional Trials (SPIRIT 2013; Additional file 1) [14] and the Consolidated Standards for Reporting of Trials CONSORT guidelines [16]. Mothers who consent to participate in the study will be randomly assigned to the intervention (fresh milk) group or the control group. In the fresh milk group, mothers will be asked to provide at least two feeds of fresh milk (i.e., within 4 h of milk expression) per day. In the control group, mothers will follow the usual protocol for the collection, freezing, and use of human milk, and the current NICU standards of human milk handling and feeding will apply.

### Study population

The study population will include infants born at < 30 weeks’ gestation who never received infant formula, are admitted to one of the 29 participating tertiary NICUs in China, have parents who consent to the study, and have mothers who are willing to provide at least two feeds of fresh milk daily, 7 days a week until the infant is 32 weeks’ corrected age. Infants with major congenital anomalies or receiving palliative care, infants with mothers who are too ill to provide fresh human milk in the first week postpartum or are unable to provide at least two feeds of fresh milk a day, infants with congenital *Cytomegalic virus* (CMV) infection, and infants whose mothers have blood CMV-IgM (+) or CMV-DNA (+) or are unwilling to consent will be excluded. Congenital CMV-infection is defined as positive CMV NAT (or positive viral culture obtained from clinician-ordered testing) in blood or urine within 2 weeks of life [20].

China is a large country with four municipalities, 23 provinces, and five autonomous regions, and we were able to recruit 29 tertiary NICUs of the same academic level from 18 provinces, municipalities, or autonomous regions to participate in our study. All hospitals have milk banks, are located in cities of a similar size with above-average economic status and similar patient mix, and admit on average an estimated 2397 infants per year who would be eligible for the study [Siyuan Jiang, MD, written communication, October 2018]. The following hospitals have committed to participate in the RCT: Children’s Hospital Affiliated to Zhengzhou University, Children’s Hospital of Fudan University, the Affiliated Obstetrics and Gynecology Hospital of Nanjing Medical University and Nanjing Maternity and Child Health Care Hospital, Children’s Hospital of Nanjing Medical University, Guizhou Maternity and Child Health Care Hospital, Shanghai First Maternity and Infant Hospital, The Women and Children’s Health-Care Hospital of Hubei Province, Henan Provincial People’s Hospital, Northwest Women and Children Hospital, The First Affiliated Hospital of Zhengzhou University, Shenzhen Maternity and Child Healthcare Hospital, Maternal and Child Health Hospital of Guangxi Zhuang Autonomous Region, Xinjiang Uiger Municipal People’s Hospital, Children’s Hospital of Soochow University, Women’s Hospital School of Medicine Zhejiang University, Qilu Children’s Hospital of Shandong University, Xiamen Children’s Hospital, Children’s Hospital School of Medicine Zhejiang University, The Third Affiliated Hospital of Zhengzhou University, Woman and Children Hospital of Zhengzhou, Anhui Provincial Hospital, Xianmen Humanity Hospital, Children’s Hospital of Capital Institute of Pediatrics, Guangdong Women and Children Hospital, Jiangxi Children’s Hospital, First Affiliated Hospital of Xinjiang Medical University, Shanghai General Hospital and Shanghai Jiaotong University, Gangdong Maternal and Child Health Care Hospital, Guangzhou Women and Children’s Medical Center, and Children’s Hospital of Hainan Province.

### Enrollment

**Fig. 1:**
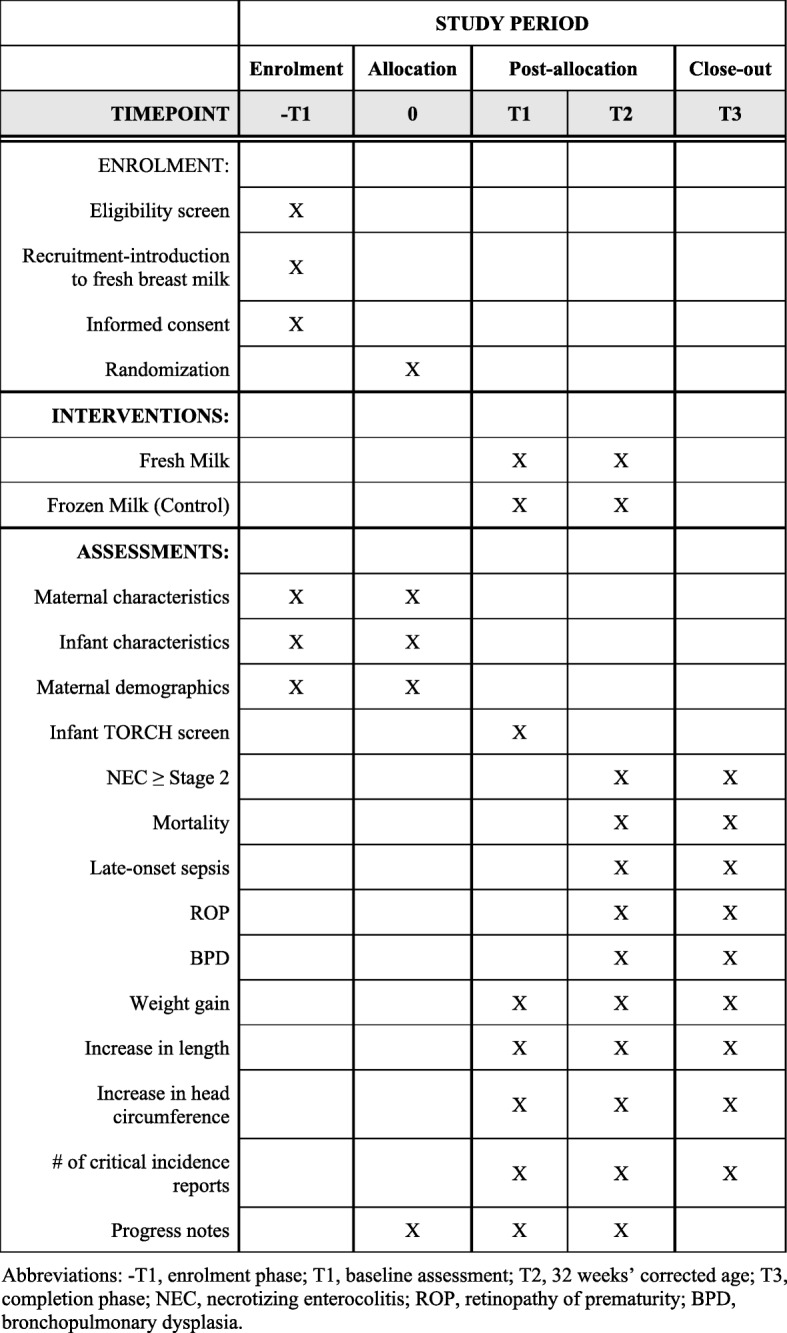
SPIRIT 2013 Schedule of infant enrollment, intervention, and assessment. Abbreviations: *-T1* enrollment phase, *T1* baseline assessment, *T2* 32 weeks’ corrected age, *T3* completion phase, *NEC* necrotizing enterocolitis, *ROP* retinopathy of prematurity, *BPD* bronchopulmonary dysplasia

Clinicians will approach eligible mothers for consent at their first visit to the NICU following their infant’s admission. The clinicians will explain the study verbally and deliver printed information sheets describing the purpose and process of the study (Fig. 1). Enrollment will occur before the infant’s first oral feed and after the parents give consent (Additional file 2). Infants whose parents decline to consent will receive standard care.

### Feeding intervention

#### Standard feeding and fortification procedures

Infants born at < 30 weeks’ gestation are initially provided with nutrition intravenously (total parenteral nutrition [TPN]). If the infant is not suffering from any condition that affects the function of the gastrointestinal system, oral feeds are introduced as early as possible. Human milk is introduced into the stomach through a nasogastric tube according to a schedule that is dependent on the infant’s weight at birth. The amount of milk is very small at first, then it is gradually increased if the infant tolerates the milk well. The frequency of feeding varies, but it is most often every 2 or 3 h, depending on the weight of the infant. As the volume of milk increases, the volume of TPN is decreased until the infant receives all their nutrition from human milk.

Once the infant’s total nutrition is from human milk (“full enteral feeds” is defined as 120 ml/kg/day), fortifiers and other nutritional supplements are added to each feed. The first supplement added is human milk fortifier; 4 days later, extra protein is also added; and then after another 3 days, vitamins and iron are added to each feed (Additional file 2).

Mother’s own milk is the food of choice, followed by human donor milk. The decision to supplement the mother’s own milk with donor milk is dependent on the mother’s ability to produce milk in sufficient quantity for all feeds. Often in the first few days of life preterm infants are fed donor milk as the mother may not start producing milk right away or may still be recovering from the birth. Mothers may also produce less milk than is needed, either initially or throughout the breastfeeding period, so some feeds may be supplemented with donor milk.

Current standard procedure dictates that in every case where expressed human milk is used, the milk is collected from the mother and frozen in the milk preparation room. Each evening the feeds for the following day are ordered, and milk is defrosted and prepared. Infants move from enteral feeds to breastfeeding when they are clinically judged to be able to do so.

#### Fresh milk feeding and fortification procedure

The exact same standard feeding and fortification procedures (timing of initiation, amount, frequency, and supplementation with human donor milk as needed) will be followed for infants in the fresh milk group, with the one exception that at least two feeds per day will be fresh milk until the infants are 32 weeks’ corrected age. “Fresh” milk is defined as human milk that has not been frozen, chilled, or pasteurized and has been expressed less than 4 h prior to feeding the infant. The 4-h time window allows us to retain live human milk cells that have a 4-h half-life.

#### Human milk collection

In both the control and fresh milk groups, all mothers will be present in the NICU to pump milk for a “fresh” feed at least two times per day, 7 days a week, using the Medela Symphony breast pump. Human milk will be expressed into a sterile plastic container as per standard NICU protocol. The container will be labeled with the mother’s bar code and immediately given to a research nurse dedicated to the implementation of this study. The research nurse will take the milk to the milk preparation area where the details of the milk and feeds will be entered into a database, and then she will prepare the milk and deliver it to the NICU, where the infant’s nurse will feed the infant. Milk being prepared for a fresh feed will not be heated but will be kept at room temperature at all times.

Any additional milk expressed by the mother in the NICU or at home that exceeds the 4-h fresh-milk window will be transferred to a sterile container, frozen, and stored in the milk preparation room for use when the mother is not available to provide fresh milk. Human milk collection and handling of previously frozen milk will follow standard NICU protocol. Mothers who provide fewer than 2 feeds of fresh milk every day will remain in the study per intention-to-treat principles. Furthermore, retaining mothers who do not produce enough milk will enable us to calculate the percentage of mothers who can produce at least two feeds of fresh milk a day, which in turn, allows us to determine the feasibility of standard fresh milk guidelines.

Mothers in both the control and fresh milk groups will continue to provide human milk in the manner described from the time the infant is able to tolerate enteral feeds until the infant is 32 weeks’ corrected age. Mothers will also be encouraged to express human milk when they are away and bring it into the NICU, as per standard NICU protocols. In cases where a mother is not able to produce enough human milk for all her infant’s feeds, the remaining feeds that are not being provided as fresh or frozen mother’s own human milk may be supplemented using human donor milk.

### Safety

**Fig. 2:**
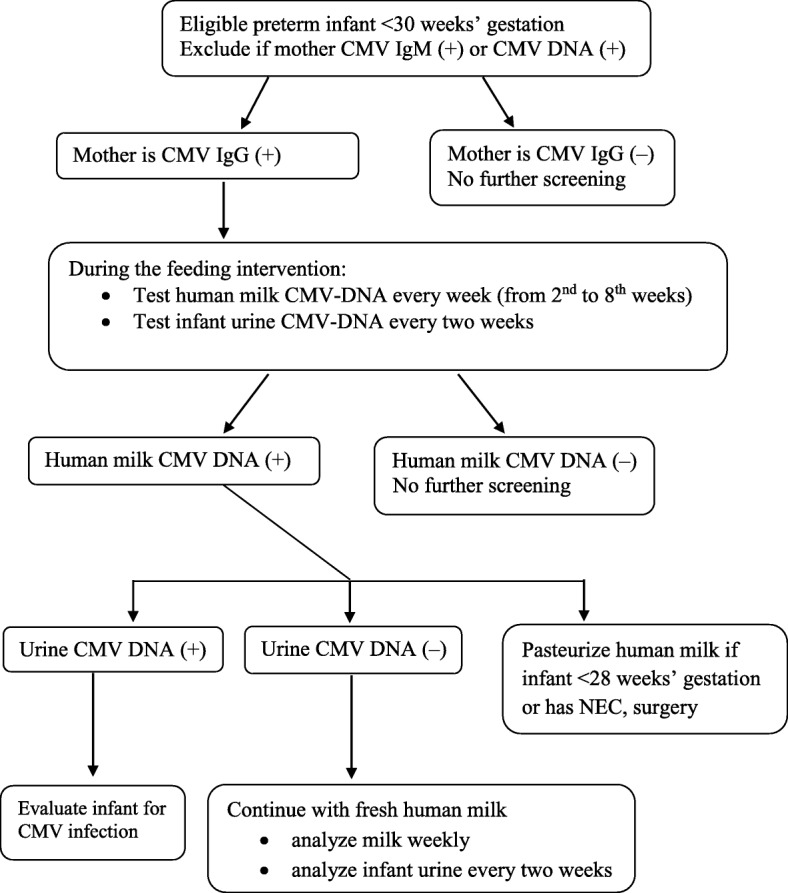
CMV infection monitoring during human milk feeding

Every neonate admitted to the NICU will complete the TORCH screen for toxoplasma, rubella, cytomegalovirus, and herpes simplex, and mothers will be asked about CMV during screening. During the fresh milk feeding, human milk will be monitored for CMV, and CMV infection associated with human milk will be managed (Fig. 2) [20–23]. If an event occurs where the standard practice requires stopping feeds, such as NEC, the feeding protocol will be stopped for that patient.

All cases of mortality, NEC, sepsis, and critical incidence reports (including infants receiving the wrong milk, infection that is related to feeds, delays in milk preparation that result in missed feeds, and any other incident clinicians deem concerning) that occur in infants enrolled in the study will be critically reviewed by a Data Safety and Monitoring Committee; the study will stop if safety concerns are raised. Stopping criteria and decisions will be decided by the Data Safety and Monitoring Committee. The Data Safety and Monitoring Committee will consist of the following three people who are experts in the use of human milk to feed infants born preterm and research methodology:

1. Dr. Yang Yi, Director of Research, Children’s Hospital of Fudan University, Shanghai, China
2. Dr. Chen Chao, Director of Neonatology, Children’s Hospital of Fudan University, Shanghai, China
3. Dr. Weili Yan, Statistician, Children’s Hospital of Fudan University, Shanghai, China

### Outcomes measures

The primary outcome of the study will be a composite of mortality or NEC ≥ stage 2. The secondary outcomes of the study will be mortality before discharge; NEC ≥ stage 2; NEC needing surgery; late-onset sepsis; retinopathy of prematurity (ROP) requiring treatment; bronchopulmonary dysplasia (BPD); change in weight, length, and head circumference; change in weight, length, and head circumference z-scores; time to full enteral feeds (160 mL/kg/day); and number and type of critical incident reports, including feeding errors. NEC will be defined according to Bell’s classification [24]. Sepsis will be defined as isolation of bacterial, fungal or viral organism from blood or cerebrospinal fluid in a symptomatic infant. ROP will be defined according to the International Committee for the Classification of Retinopathy of Prematurity [25]. BPD will be defined as oxygen need at 36 weeks’ postmenstrual age [26]. Weight, weight z-score, length and head circumference will be assessed on a weekly basis until the infant is 9 weeks (63 days) of age.

### Randomization and blinding

A blocked randomization method stratified by NICU will be used to assign infants to either the control or fresh milk groups. Infants from multiple-birth pregnancies will be randomized to the same group. We will use an online software application (https://www.graphpad.com/quickcalcs/randomize1.cfm) to generate the allocation scheme and randomize patients electronically. If the parents wish to withdraw consent at any time, all study procedures will cease, and the infant will be fed following the current standard procedures for human milk handling and feeding as directed by the clinical team.

The study will be double blinded; thus, the medical and nursing teams caring for the infants will be unaware of the type of human milk used for feeding. A research nurse will label the milk with only the study site and infant number and deliver the milk to the nurse caring for the baby, who will be blinded to whether the baby is receiving fresh or frozen human milk. The outcomes of the infants will be collected by an investigator blinded to the group allocation. Data analysis will be performed using a binary treatment code to maintain group allocation of blinding until the results are finalized. No unblinding will occur in this study.

### Timeline

#### Preparation (July 1, 2018, to December 31, 2018)

During the preparation phase, we will develop and implement an education program for staff [19] regarding the study protocol, which will include in-service sessions held before the study period begins. Furthermore, we will obtain approval from the research ethics boards as required for the study protocol, and then we will implement the required changes to unit feeding policies (Fig. 1, Additional file 3).

#### Enrollment (January 1, 2019, to December 31, 2020)

The enrollment phase of the study will continue until 1549 infants and their mothers have completed the fresh milk protocol and 1549 infants and their mothers have completed the control protocol. Data collection will begin when the first infant is enrolled and will be completed when the last infant has reached 32 weeks’ corrected age.

#### Project completion (January 2, 2021, to June 30, 2021)

During the project completion phase, any outstanding data will be collected and entered, data will be cleaned up and analyzed, and the study results will be reported.

### Data collection

We will collect data from January 1, 2019, to December 31, 2020, on maternal and infant characteristics including demographics, risk factors, delivery complications, illness severity scores, infant complications, weight, growth, and daily feeding practices, including probiotics. All information will be collected on data forms, with data being entered into an encrypted excel file database for subsequent analysis. Study-specific variables will include compliance with the fresh milk protocol, the number and volume of fresh milk feeds provided to study infants on each day, and the response of the infant following the feed (Additional file 4). Outcomes will be assessed at 32 weeks’ corrected age. For completion and to allow collection of outcomes that can only be assessed beyond 32 weeks (e.g., BPD), outcomes will also be collected at discharge. For infants discharged before 32 weeks’ corrected age, we will follow up until they reach 32 weeks’ corrected age and record data on the primary and secondary outcomes. All data will be de-identified, and we will use patient numbers instead of names when summarizing data. Data access will comply with privacy regulations, and all data will be kept by a specialist to protect the patients’ privacy.

The Research Institute of Children’s Hospital of Fudan University in Shanghai, China, will host the coordinating center for the Fresh Milk study. The coordinating center will store all the study data and complete data analysis. Dr. Huiqing Sun from the Children’s Hospital affiliated to Zhengzhou University will be the Principal Investigator of the study and will be responsible for registering, receiving ethical approval, and notifying and receiving approval for changes to the study from the regulatory bodies. Central ethical approval was confirmed from the ethics committee of the Children’s Hospital affiliated to Zhengzhou University for Clinical Research (REB #2018042701, Additional file 3), and we will not begin recruiting at other centers in the trial until local ethical approval has been obtained. The study is registered in the Chinese Clinical Trial Registry (www.chictr.org.cn) #ChiCTR1900020577. To ensure ethical standards are met, we will obtain informed consent from each participating parent before enrollment and will respect the parents’ decision to join or leave the study at any time. Furthermore, we will use an incident reporting system that will be completed within 24 h of an incident and will include a formal written report to protect the patients’ safety. Finally, we will obtain the parents’ written approval for publishing their names or pictures on the research update in addition to following the data privacy procedures described above.

### Sample size

The sample size justification was based on the primary outcome and the hypothesis. A recent quality improvement study found that the incidence of the composite outcome mortality or NEC ≥ stage 2 was 29% in 25 Chinese NICUs [Siyuan Jiang, MD, written communication, October 2018]. Assuming a primary outcome rate of 29% for the control group, we estimated that a sample size of 3098 (1549 patients per arm) will achieve an 80% power to detect at least a 16% relative reduction in the incidence of the primary outcome in the fresh milk group when compared to the control group. With an average of 2397 eligible infants admitted to participating hospitals each year and an estimated enrollment rate of 60%, we expect to recruit 1438 infants per year and the study to take 2 years. We used the chi-square test for the power analysis, assuming a significant level of 0.05. Therefore, the proposed enrollment of 3098 patients will have sufficient power to test our hypothesis taking into account a possible 5% data loss to follow-up.

### Statistical analysis

Data from the study will be analyzed on an intention-to-treat basis. Patient characteristics at baseline will be summarized within each of the groups using the descriptive statistical methods suggested by the CONSORT statement for randomized clinical trials. To examine the difference in the rate of the primary outcome between the control and fresh human milk groups, we will use the chi-square test. We will compare the secondary outcomes of the control and fresh milk groups using the chi-square test or Fisher exact test, as appropriate, for categorical variables and the Student T test or Wilcoxon Rank Sum test, as appropriate, for continuous variables.

As secondary analyses, we will also, if applicable, determine whether a variation exists in the treatment effect across the centers/NICUs by presenting the effects by individual centers/NICUs graphically. If variation is observed, we will further compare the binary outcomes using the Cochran-Mantel-Haenszel test to account for the effect of center, as well as conducting fixed-effect logistic regression (for binary outcomes) or linear regression (for continuous outcomes) without including treatment-center interaction term. The fixed-effect model including treatment-center interaction term will be further applied to examine the heterogeneity of the effect across centers when the variation in the center-effect is identified. If variation exist in the center-effect but no heterogeneity of the effect is observed, a marginal logistic or linear regression model with generalized estimating equations (GEE) approach will be applied to estimate the main treatment effect to account for the center effect. If the heterogeneity of the effect is observed, subgroup analysis stratified by the homogeneous centers using marginal models with GEE approach will be applied if applicable.

To examine whether a variation exists in the treatment effect across gestational age group (i.e., GA < 28 vs GA: 28–30), birth weight group (i.e., BW < 1000 g vs BW: 1000-1500 g) or upon receipt of probiotics, fixed-effect logistic or linear models will be used that include GA (or BW or probiotic) group and the interaction between treatment and GA group (or BW or probiotic), if applicable.

In China, approximately 17% of infants born preterm die at home following discharge against medical advice [Siyuan Jiang, MD, written communication, October 2018]. Since we are using intention-to-treat analysis in our study, these infants will be included in our analysis. However, we will perform a subgroup analysis that only includes infants who were actively treated, which includes 1991 infants per year on average [Siyuan Jiang, MD, written communication, October 2018]. The primary outcome rate is 17%, on average, for infants who are actively treated; therefore, we estimate that a total sample size of 2292 infants (1146 per arm) will achieve an 80% power to detect at least a 25% relative reduction in the incidence of the primary outcome in the fresh milk group when compared to the control group.

We will also apply linear or nonlinear mixed-effect models for the longitudinal secondary outcomes (weight or change in weight z-score over time) to compare the rate of the change in the outcomes between the two groups, adjusted for the infant- and NICU- level characteristics if applicable. For secondary outcomes, no adjustment for multiple comparisons will be conducted.

For the primary outcome, to minimize possible bias due to drop out or missing data, we will also, if applicable, conduct logistic regression adjusted for propensity score estimated based on the infant and NICU characteristics available. A two-sided *p* value of < 0.05 will be used to determine the statistically significant difference.

## Discussion

Human milk provides the optimal nutrition as well as all the protective immune and developmental components needed for healthy infant development. The benefits of feeding preterm infants human milk are well-documented [27] and include decreased incidences of NEC, late-onset sepsis, feeding intolerance [6, 28], and BPD [27]. Our fresh milk study will evaluate the impact of feeding infants born at < 30 weeks’ gestation with fresh milk, within 4 h of its expression, on NEC ≥ stage 2 and on secondary outcomes, including sepsis, ROP, and BPD.

Human milk has many nutrients and bioactive factors that are beneficial to both term and preterm infants. Carbohydrates, particularly human milk oligosaccharides (HMO), are important for protection against infection, particularly NEC [29]. Furthermore, a multitude of proteins are present in human milk that form its essential nutrition and also play contributory roles in infant immune protection as well as the development of the immune system and the gut [30, 31]. Lipids come in many different forms and provide a major source of energy found in human milk [32]. Fatty acids, a type of lipid, are important for brain development [33] and can inactivate pathogens *in vitro* [34]. Finally, other bioactive components, including miRNAs [35, 36], stem cells, and microbes [37, 38], are present in human milk and are involved in immune response to infection and various aspects of development [37, 39–41].

Currently, the standard NICU procedure for fresh milk expressed by mothers of preterm infants is to freeze it and sometimes pasteurize it, depending on where the mother expressed it, before feeding. However, many components of human milk change with freezing, pasteurizing, and subsequent reheating. While carbohydrates remain relatively intact [42], bioactive proteins, lipids, and energy content are reduced after these steps of milk processing [﻿11﻿, ﻿43, 44]. In addition, following freezing, human milk shows lipid peroxide formation [45, 46]. Finally, no immune cell or stem cell activity is present following pasteurization or freezing or heating [﻿43﻿, 47].

*Cytomegalic virus* infection acquired from fresh human milk has been discussed as a potential safety concern in preterm infants [48]. However, the issue is controversial, and guidelines vary [49]. Some guidelines recommend that preterm infants are fed pasteurized mother’s own milk because of the potential risk of contamination by CMV. However, this may be unnecessary considering that if a mother is CMV positive, the infant already will have been exposed to CMV *in utero*. We will address this concern in our study by requiring every neonate admitted to the NICU to be tested for CMV and pasteurizing infected milk before use.

Oxidative stress is a major problem for ill infants and for preterm infants in particular; therefore, energy intake and antioxidant defense are crucial for preterm infants’ wellbeing [﻿45﻿]. Very preterm infants have diminished antioxidant capacity and are frequently exposed to oxidative stress from multiple sources such as infection, oxygen therapy, and mechanical ventilation. Furthermore, excess oxygen can secondarily lead to retinopathy of prematurity or chronic lung disease in preterm infants [﻿45﻿]. NEC, intraventricular/periventricular hemorrhage, and retinopathy of prematurity are all thought to be consequences of the imbalance between antioxidant capacity and oxidative stress [50]. Previous studies reported that human milk has important and essential antioxidant components, which may prevent and protect against diseases in infancy [45]. Human milk feeding remains an essential tool to help in the protection against free radicals, oxygen reactive species and oxidative stress, with fresh human milk having been reported to have the highest antioxidant activity [﻿45].

Our study will have some limitations. One limitation is a potential drop-out rate of 10% of enrolled families (based on our pilot study data) [﻿19﻿] due to factors such as time constraints. To minimize the potential bias from families leaving the study, we will use intention-to-treat analysis. If applicable, we will also conduct regression adjusted for propensity score estimated based on the infant and NICU characteristics available. Last, in China, approximately 17% of infants born preterm per year are discharged against medical advice and die at home. These infants will be included in our intention-to-treat analysis. To address this concern, we will perform a subgroup analysis of the primary outcome that only includes infants who were actively treated.

This study will be the first large, multicenter, prospective RCT to associate neonatal outcomes when feeding fresh human milk to preterm infants < 30 weeks’ gestation. Importantly, the results will determine the efficacy of fresh mother’s own milk to improve neonatal outcomes. The data of the study will be of significant interest to neonatal medical centers around the world, particularly because of the potential for fresh human milk to be a cost-effective way to improve and potentially optimize preterm infant care and outcomes.

## Trial status

The current protocol version is 4 on October 2, 2018. Patient recruitment began January 1, 2019, and should be completed by December 31, 2020.

## Supplementary information

**Additional file 1.** SPIRIT 2013 Checklist.

**Additional file 2.** Consent Form.

**Additional file 3.** Research Ethics Board Approval.

**Additional file 4.** Data Forms.

## Data Availability

Investigators and the coordinating center will have access to the data for analysis and publication purposes. After publication, the datasets will be available from the corresponding author on reasonable request.

## Abbreviations

CMV: cytomegalic virus
DNA: deoxyribonucleic acid
g: grams
IgG: immunoglobin G
NEC: necrotizing enterocolitis
NICU: neonatal intensive care unit
TPN: total parenteral nutrition
