# Supplementary material for: A randomized controlled trial protocol comparing the feeds of fresh versus frozen mother’s own milk for preterm infants in the NICU
Source: Trials. 2020 Feb 11;21:170. doi: 10.1186/s13063-019-3981-4 (PMC7014600; doi:10.1186/s13063-019-3981-4)
Supplement: Supplementary file 2 — Additional file 2. Consent Form. [file 13063_2019_3981_MOESM2_ESM.docx]

**Additional file 2: Consent Form**

# A randomized controlled trial to compare feeding preterm infants<30 weeks’ gestation fresh versus frozen mother’s own milk in the NICU ——Informed Consent Form

Dear Parents,

This is a multicenter study to evaluate the impact of feeding infants born at <30 weeks’ gestation fresh, unprocessed, mother’s own milk within 4 hours of expression on the primary outcome, the composite mortality or necrotizing enterocolitis (NEC) ≥ stage 2. There are 28 level 3 hospitals will participating in this study. We ask each parent to sign this release form after fully reading, understanding and agreeing to the terms of the study. Please be assured that your child will receive the highest standard of care regardless of your participation in this study.

**Title of study**

# A randomized controlled trial to compare feeding preterm <30 weeks’ gestation infants fresh versus frozen mother’s own milk in the NICU: a study protocol

**Primary Investigator**

Huiqing Sun, Director of Neonatal Department (preterm infant intensive care unit), Children’s Hospital of Zhengzhou University

**Mentor of the study**

Shoo Kim Lee, Canadian Institute of Health Research (CIHR)

**Duration of study**

From January 1, 2019 to December 31, 2020

**Background**

Mother’s breast milk is best for preventing necrotizing enterocolitis (NEC) in premature infants. It contains bioactive components including growth factors, anti-inflammatory factors, hormones, and many active cells, which give breast milk antimicrobial and anti-inflammatory properties. More recently, breast milk was found to be a rich source of pluripotent stem cells, which might benefit the infant through growth or regeneration. The evidence strongly suggests that fresh breast milk may have a protective effect against both infection and NEC. In many NICUs in China, breast milk is routinely frozen or pasteurized to avoid the possibility of infection via breast milk feeding. However, this process reduces the nutritional components and damages the protective factors and live cells. As stem cells in the milk have a half-life of 4 hours, refrigeration of breast milk for more than 4 hours will significantly reduce the amount of stem cells. Pasteurization or thawing frozen breast milk will kill all stem cells. Our hypothesis is that fresh breast milk, given within 4 hours of expression, at least once a day, will reduce the occurrence of NEC in very premature infants. We conduct this study to evaluate the feasibility and safety of fresh breast milk use in very premature infants, and to observe its potential to reduce NEC in this population.

**Introduction of the study**

The objective of this study is to evaluate the impact of feeding infants born at <30 weeks’ gestation fresh unprocessed milk within 4 hours of expression on the primary outcome, the composite of mortality or NEC ≥ stage 2. The secondary outcomes of the study will be mortality before discharge; NEC ≥ stage 2; NEC needing surgery, late-onset sepsis; retinopathy of prematurity (ROP); bronchopulmonary dysplasia (BPD); weight gain, change in weight, increase in length, increase in head circumference; time to full enteral feeds; and number and type of critical incident reports, including feeding errors. The study group will enroll 3098 infants who will receive either standard care or their mother’s fresh breast milk, which is defined as breast milk fed within 4hours after expressing at least twice a day. This will continue until the infant reaches the corrected age of 32 weeks. The mother is encouraged to supply exclusive breast milk feeding to her baby; donor human milk is supplemented as mother’s breast milk is in shortage.

**Benefits of participation**

1) Your child will get the highest quality of breast milk, and fresh milk may promote maturation of the intestines and infant growth, reduce NEC and infection, and may help the development of brain. It may be beneficial for the short-term and long-term outcomes of premature infants.

2) You may communicate with the staff that taking care of your baby more frequently.

3) You will be able to learn more about the details of your baby’s feeding and growth.

4) Participation may help mothers to produce enough breast milk, to recover from delivery, and to promote breast feeding after discharge.

5) You and your baby’s contribution to this study may help to promote the spread of breast milk feeding in China.

**Risks of participation**

1) Similar to other clinical studies, uncertainties may exist with respect to the guidelines and procedures.

2) If the mother has an infection such as HIV, CMV, HPSV, TB, or if breast milk is contaminated, raw fresh milk may cause the transmission of pathogens to the baby. If mother has any symptoms of infection, including fever, diarrhea, or rash, please consult the doctor to make a decision on whether your breast milk should be fed to the baby.

3) If a Mother is CMV IgG positive and CMV-IgM negative, during the fresh milk feeding, human milk will be monitored for CMV and CMV infection associated with human milk will be managed.

The fresh breast milk CMV-DNA will be tested every week from 2^nd^ to 8^th^ weeks after birth. The infant urine CMV-DNA will be tested every two weeks.

If breast milk CMV- DNA test is negative, no further screening.

If breast milk CMV-DNA is positive, and with infant urine CMV-DNA positive, the infant will be evaluated for CMV infection.

If breast milk CMV-DNA is positive, and with infant urine CMV-DNA negative, the infant will be continued in trial, and analyze milk weekly and analyze infant urine every two weeks.

If breast milk CMV-DNA is positive, and the infant is <28 weeks’ gestation, has NEC, or needs surgery the breast milk will be pasteurized.

If the Mother is CMV-IgG negative, no further screening.

4) To ensure the highest quality of fresh breast milk, mothers should keep their bodies clean, take regular showers, and cut fingernails regularly. The hospital will supply you with a breast pump and containers. Mothers will be supervised when cleaning their hands and using the pumps.

5) Mothers will be required to come to the hospital every day to pump breast milk.

**Privacy protection**

1) Access to any information related to you or your child will only be available to the researchers involved in the study.

2) The hospital will provide a private pumping area for your comfort and privacy.

3) Clinical materials including photos and pictures of you and your child will be collected for data analysis, and may be used for academic exchange during academic meetings. We will obtain your consent if we use you or your child’s photos. These materials will not be used for any commercial purposes.

**Cost**

This study is financed by the Chinese Neonatal Network and the Canadian Institutes of Health Research. This study has been approved by medical ethics committee. To protect the rights and interests of enrolled parents and babies and to avoid potential study bias, parents will not be charged for any extra services, nor will they receive payment for enrollment.

**Withdrawal**

**You have the right to withdraw from the study at any time.**

**Extra Information**

1) You can bring the information package home and discuss with your family before you make a decision.

2) Going outside after delivery will not do any harm to mothers. Mothers should avoid fatigue and stress while maintaining a well-balanced diet, which will be beneficial for mothers to recover from delivery and to produce enough breast milk.

3) If at any time you have questions or concerns regarding the study please do not hesitate to contact us. Our telephone number is 86-0371-85515912

Thank you for your participation! Your participation will help to improve quality of care for high-risk premature infants. Let us work together to create a better future!

Doctor’s name and title

Meeting location

Date

Please select one of the following options

I fully understand the purposes, contents, and potential benefits and risks of this study. I choose to participate in this study with my baby of my own free will.

I decline to participate in this study.

baby’s name

parent’s name

parent’s signature

date

parent’s telephone number：
