# Supplementary figures and images for: A randomized controlled trial protocol comparing the feeds of fresh versus frozen mother’s own milk for preterm infants in the NICU
Source: Trials. 2020 Feb 11;21:170. doi: 10.1186/s13063-019-3981-4 (PMC7014600; doi:10.1186/s13063-019-3981-4)

**Additional file 3: Research Ethics Board Approval**

**
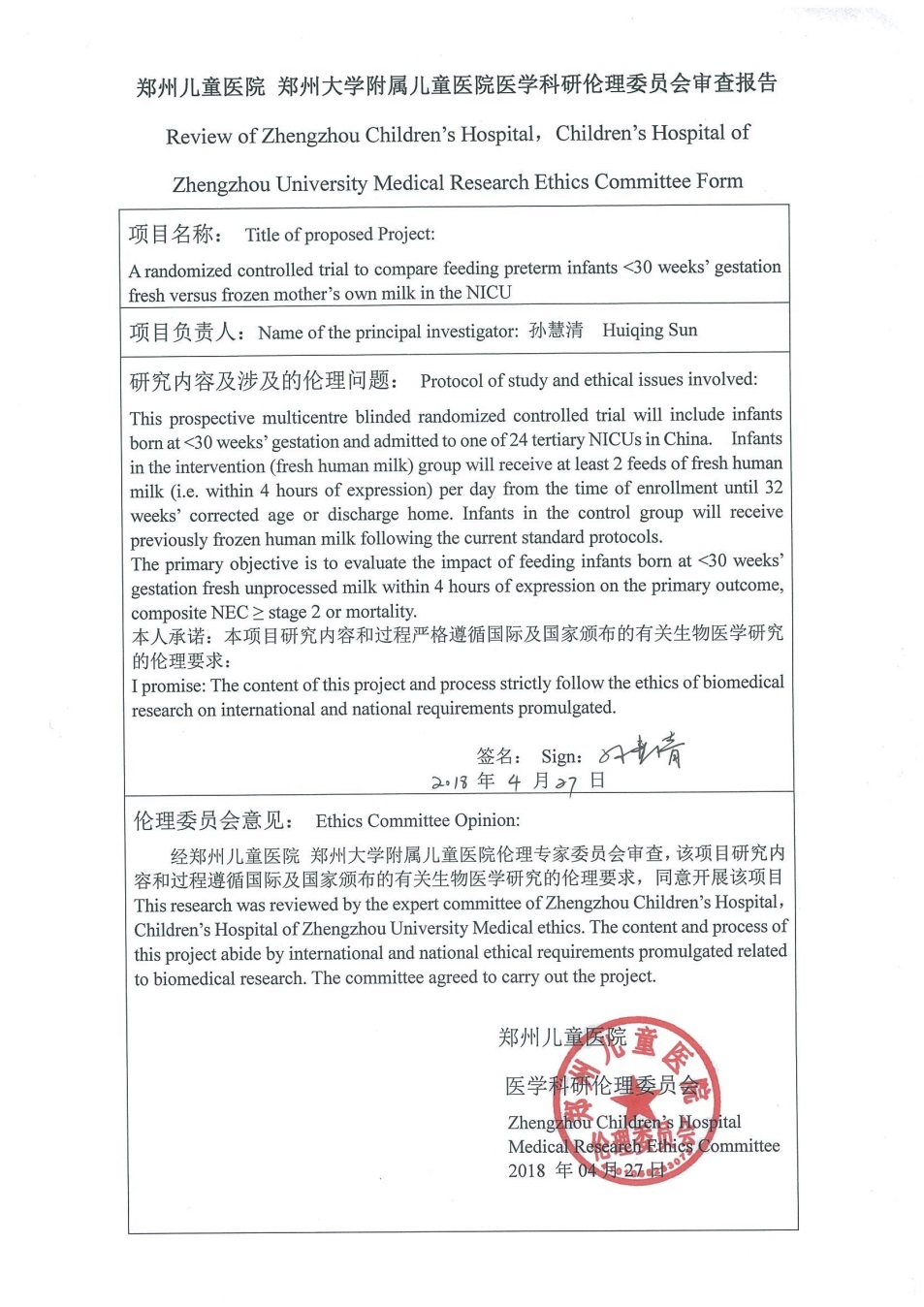
**

Supplement: Supplementary file 3 — Additional file 3. Research Ethics Board Approval. [file 13063_2019_3981_MOESM3_ESM.docx]
