# Supplementary material for: A randomized controlled trial protocol comparing the feeds of fresh versus frozen mother’s own milk for preterm infants in the NICU
Source: Trials. 2020 Feb 11;21:170. doi: 10.1186/s13063-019-3981-4 (PMC7014600; doi:10.1186/s13063-019-3981-4)
Supplement: Supplementary file 4 — Additional file 4. Data Forms. [file 13063_2019_3981_MOESM4_ESM.docx]

**Additional file 4: Data Forms**

**SECTION A: ENROLLMENT**

1. Enrollment date: ________________ (YYYY/MM/DD)
2. Age of infant at enrollment: ____________ (hours)

**SECTION B: MATERNAL DATA**

1. Mother’s date of birth: ___________________________ (YYYY/MM/DD)
2. Gravida: ___________ Para: _______________ Abortions: ___________
3. Pre-pregnancy Weight: ________________ (kg)
4. Marital status:
5. Married or common law
6. Divorced or separated
7. Widowed
8. Single
9. Highest level of education:
10. Elementary school
11. High school
12. Trade certificate or apprenticeship
13. Professional registration (non-degree)
14. College diploma
15. University degree

| **Conditions / Interventions** | **Yes**  **✓** | **No**  **✓** | **Unknown**  **✓** | **Please specify** |
| --- | --- | --- | --- | --- |
| Conception by assisted reproductive technology |  |  |  |  |
| Smoke or use illicit drugs during pregnancy |  |  |  |  |
| Antenatal corticosteroid |  |  |  |  |
| **Conditions / Interventions (continued)** | **Yes**  **✓** | **No**  **✓** | **Unknown**  **✓** | **Please specify** |
| Gestational diabetes |  |  |  |  |
| Hypertension or Pre-eclampsia |  |  |  |  |
| MgSO4 during labour |  |  |  |  |
| Clinical chorioamnionitis |  |  |  |  |
| Antenatal bleeding |  |  |  |  |
| Postpartum haemorrhage |  |  |  |  |
| Depression / Anxiety |  |  |  |  |
| Previous breast surgery |  |  |  |  |
| Allergies |  |  |  |  |
| Other conditions |  |  |  |  |

1. Other, specify ________________________________________
2. Maternal medical conditions and interventions
3. Length of ROM
4. <24 hours
5. 24 hours to 1 week
6. >1 week
7. Unknown
8. Labor initiation
9. No labour
10. Spontaneous
11. Augmented
12. Induced
13. Unknown
14. Mode of delivery
15. Vaginal
16. Assisted vaginal
17. Emergency Caesarean
18. Elective Caesarean
19. Unknown
20. Presentation
21. Vertex
22. Breech
23. Other
24. Unknown

**SECTION C: INFANT DATA**

1. Infant’s date of birth: _____________________________ (YYYY/MM/DD)
2. Infant’s time of birth: ________________ (24 hr)
3. Infant’s sex
4. Male
5. Female
6. Ambiguous
7. Unknown
8. Gestational age at birth: ____________________________
9. Apgar Scores at
10. One minute: _____________
11. Five minutes: ____________
12. Ten minutes: ____________
13. Number of births in this pregnancy: ____________

Birth order: ______________

1. Admission status:
2. Inborn
3. Outborn
4. Respiratory Support

| **Type of Respiratory Support** | **Number of Days** | **End Date (YYYY/MM/DD)** |
| --- | --- | --- |
| CPAP |  | N/A |
| Mechanical Ventilation |  | N/A |
| Oxygen   1. At 28 days __________ (%) 2. At 36 weeks CGA ____________ (%) |  |  |

1. Surfactant administration
2. Yes, specify type __________________ and date ______________ (YYYY/MM/DD)
3. No
4. Caffeine administration
5. Yes, specify start date _________________________ (YYYY/MM/DD)
6. No
7. TPN
8. Yes, specify number of days _______ and end date_____________ (YYYY/MM/DD)
9. No
10. Probiotics
11. Yes, type: Combined Clostridium Butyricum and Bifidobacterium Powders, or Compound Eosinophil-Lactobacillus Tablets
12. No
13. NEC diagnosis
14. Yes, specify stage _______ and date of first episode ___________ (YYYY/MM/DD)
15. No

NEC treatment

1. Surgical
2. Peritoneal drainage
3. No

Multiple episodes

1. Yes
2. No

Acquired stricture

1. Yes
2. No
3. Gastrointestinal Perforation
4. NEC related
5. Spontaneous, specify SIP treatment
6. Surgical
7. Peritoneal drainage
8. None
9. Was ROP screening done
10. Yes
11. No

ROP diagnosis

1. Yes, specify highest stage _______________
2. No

ROP treatment

1. Laser
2. VEGF blockers
3. Other surgery
4. None
5. IVH diagnosis
6. Yes, specify grade _____________________
7. No
8. PVL (with cyst development confirmed by MRI)
9. Yes
10. Suspected
11. No
12. Sepsis diagnosis
13. Yes
14. No
15. Antibiotics
    1. Yes; days on antibiotics _____________________
    2. No

Positive Blood or CSF cultures

| **Date (YYYY/MM/DD)** | **Organism** | **Culture**  **(Blood or CSF)** |
| --- | --- | --- |
|  |  |  |
|  |  |  |
|  |  |  |
|  |  |  |
|  |  |  |
|  |  |  |
|  |  |  |

1. RDS diagnosis
2. Definite
3. Uncertain
4. Unknown / not available
5. No RDS
6. HIE diagnosis
7. Yes, specify stage ______________________
8. No
9. Discharge / transfer destination
10. Home
11. Died, specify cause of death ________________________________
12. Palliative
13. Another inpatient area at your hospital
14. Another hospital

Date of discharge / death / transfer: _____________________________ (YYYY/MM/DD)

1. Breast milk CMV Screening

| Test time (weeks after birth) | CMV test | Results |
| --- | --- | --- |
| 2 weeks |  |  |
| 3 weeks |  |  |
| 4 weeks |  |  |
| 5 weeks |  |  |
| 6 weeks |  |  |
| 7weeks |  |  |
| 8weeks |  |  |

| Test time (weeks after birth) | CMV test (urine) | CMV test (blood) | Results |
| --- | --- | --- | --- |
|  |  |  |  |
|  |  |  |  |
|  |  |  |  |
|  |  |  |  |
|  |  |  |  |
|  |  |  |  |
|  |  |  |  |

1. Infant CMV Screening
2. CMV diagnosis

a yes

b No

1. Incident reports

| Type of Incident | Severity | Date (YYYY/MM/DD) |
| --- | --- | --- |
|  |  |  |
|  |  |  |
|  |  |  |
|  |  |  |

**SECTION D: WEEKLY GROWTH DATA**

***… ...continue until discharge***

***Use extra pages as needed***

| Infant Age | Weight (grams) |
| --- | --- |
| Birth |  |
| 7 days |  |
| 14 days |  |
| 21 days |  |
| 28 days |  |
| 35 days |  |
| 42 days |  |
| 49 days |  |
| 56 days |  |
| 63 days |  |
| 70 days |  |
| 77 days |  |
| 84 days |  |
| 91 days |  |
| 98 days |  |
| 105 days |  |

**SECTION E: DAILY FEEDING DATA *(from day 1 of life until discharge; use as many pages as needed)***

| Day of life | |  | |  | |  | |  | |  | |  | |  | |
| --- | --- | --- | --- | --- | --- | --- | --- | --- | --- | --- | --- | --- | --- | --- | --- |
| Date  (YYYY/MM/DD) | |  | |  | |  | |  | |  | |  | |  | |
| Schedule  (feed frequency) | |  | |  | |  | |  | |  | |  | |  | |
|  | | Volume (ml) | Type | Volume (ml) | Type | Volume (ml) | Type | Volume (ml) | Type | Volume (ml) | Type | Volume (ml) | Type | Volume (ml) | Type |
| Feed | 1 |  |  |  |  |  |  |  |  |  |  |  |  |  |  |
|  | 2 |  |  |  |  |  |  |  |  |  |  |  |  |  |  |
|  | 3 |  |  |  |  |  |  |  |  |  |  |  |  |  |  |
|  | 4 |  |  |  |  |  |  |  |  |  |  |  |  |  |  |
|  | 5 |  |  |  |  |  |  |  |  |  |  |  |  |  |  |
|  | 6 |  |  |  |  |  |  |  |  |  |  |  |  |  |  |
|  | 7 |  |  |  |  |  |  |  |  |  |  |  |  |  |  |
|  | 8 |  |  |  |  |  |  |  |  |  |  |  |  |  |  |
|  | 9 |  |  |  |  |  |  |  |  |  |  |  |  |  |  |
|  | 10 |  |  |  |  |  |  |  |  |  |  |  |  |  |  |
|  | 11 |  |  |  |  |  |  |  |  |  |  |  |  |  |  |
|  | 12 |  |  |  |  |  |  |  |  |  |  |  |  |  |  |
| Notes | |  | |  | |  | |  | |  | |  | |  | |

**Instructions:**

Schedule – Enter feed frequency on a daily basis (e.g***. Q6h, Q4h, Q3h, Q2h***) or NPO or continuous feeding

Types of feed – ***Fresh Breast Milk (FBM), Non-fresh (NFBM), Donor (D), Formula (F)***

Mark an “X” if a scheduled feed is skipped and comment in “Notes” section
